# Risk for Low Pathogenicity Avian Influenza Virus on Poultry Farms, the Netherlands, 2007–2013

**DOI:** 10.3201/eid2309.170276

**Published:** 2017-09

**Authors:** Ruth Bouwstra, Jose L. Gonzales, Sjaak de Wit, Julia Stahl, Ron A.M. Fouchier, Armin R.W. Elbers

**Affiliations:** Wageningen Bioveterinary Research, Lelystad, the Netherlands (R. Bouwstra, J.L. Gonzales, A.R.W. Elbers);; GD Animal Health, Deventer, the Netherlands (S. de Wit);; Sovon Dutch Center for Field Ornithology, Nijmegen, the Netherlands (J. Stahl);; Erasmus Medical Center, Rotterdam, the Netherlands (R.A.M. Fouchier)

**Keywords:** Low pathogenicity avian influenza virus, poultry farms, spatial analysis, wild birds, waterways, viruses, the Netherlands, zoonoses, influenza, respiratory infections, LPAIV

## Abstract

Using annual serologic surveillance data from all poultry farms in the Netherlands during 2007–2013, we quantified the risk for the introduction of low pathogenicity avian influenza virus (LPAIV) in different types of poultry production farms and putative spatial-environmental risk factors: distance from poultry farms to clay soil, waterways, and wild waterfowl areas. Outdoor-layer, turkey (meat and breeder), and duck (meat and breeder) farms had a significantly higher risk for LPAIV introduction than did indoor-layer farms. Except for outdoor-layer, all poultry types (i.e., broilers, chicken breeders, ducks, and turkeys) are kept indoors. For all production types, LPAIV risk decreased significantly with increasing distance to medium-sized waterways and with increasing distance to areas with defined wild waterfowl, but only for outdoor-layer and turkey farms. Future research should focus not only on production types but also on distance to waterways and wild bird areas. In addition, settlement of new poultry farms in high-risk areas should be discouraged.

Avian influenza is a disease of birds caused by influenza A viruses. Wild birds, particularly migratory water birds, form a natural reservoir of avian influenza viruses. Influenza viruses carry 2 glycoproteins on their surface, hemagglutinin (HA) and neuraminidase (NA), and on the basis of these glycoproteins are divided into subtypes. Eighteen distinct subtypes of HA (H1–H18) and 11 NA subtypes (N1–N11) have been described. Influenza A(H17N10) and A(H18N11), however, were recently detected in bats but not in birds. Virtually all remaining combinations of HA 1–16 and NA 1–9 subtypes have been isolated from wild birds (*1*). Wild birds pose a special risk for introducing avian influenza viruses of all subtypes to poultry kept in free-range or outdoor facilities (*2*).

Avian influenza virus infections in wild birds usually are asymptomatic. Infection of poultry ranges from no disease to severe disease and up to 100% mortality (*3*). A virus that causes no or mild disease in chickens is considered a low pathogenicity avian influenza virus (LPAIV); a virus that causes high rates of death in chickens is considered a highly pathogenic avian influenza virus (HPAIV) (*4*). HPAIV outbreaks in poultry cause huge direct and indirect economic losses (*5*). Furthermore, on several occasions during the last decade, bird-to-human transmissions of H5, H6, H7, H9, and H10 virus subtypes have occurred, emphasizing the threat to public health worldwide (*6*). Every HPAIV described has belonged to H5 and H7 subtypes and, until the spread of the Asian HPAIV subtype H5N1 to other parts of the world by wild birds since 2005 (*7*), mainly emerged after LPAIV of these subtypes were introduced in poultry, particularly in chickens and turkeys (*8*). Therefore, LPAIV of the H5 and H7 subtypes is notifiable to the World Organisation for Animal Health; consequently, member states of the European Union have implemented surveillance programs (*9*). 

In the Netherlands, passive and active surveillance programs are in place. In the active serologic surveillance program, all poultry farms are tested 1–4 times a year. Frequency of sampling differs among poultry types (indoor- and outdoor-layer chickens, chicken breeders, broilers, ducks, and turkeys) and housing systems based on the supposed differences in the risk for LPAIV introduction. Except for outdoor-layers, all poultry types are kept indoors.

In a previous study (*10*), a significantly higher risk for LPAIV introduction was observed on poultry farms in Europe housing Anseriformes (duck, geese, and game birds) than on farms housing Galliformes (chicken breeders, broilers, layer chickens, and turkeys), and no significant differences were observed among Galliformes. In addition, Gonzales et al. (*11*) reported a significantly higher risk for LPAIV introduction on outdoor-layer, turkey, duck-breeder, and meat-duck farms than on indoor-layer farms in the Netherlands using surveillance data for 2007–2010. These studies (*10*,*11*) did not find differences in the risk for introduction among farms keeping chickens indoors, particularly between layers and broilers, possibly because of the limited data on positive introductions (or zero introductions) into broiler farms (*11*), which compromised the power of the comparisons. Our objective was to update the risk analysis of introduction of LPAIV infection using an extended surveillance period (2007–2013) and add spatial-environmental factors to the analysis that might explain part of the variation in LPAIV introductions on poultry farms in the Netherlands.

## Materials and Methods

### Data

We analyzed all data from the Netherlands’ surveillance program collected during January 2007–December 2013. In the Netherlands, 3 types of surveillance programs are used to detect avian influenza virus infections on commercial poultry farms: passive surveillance, early warning, and serologic monitoring.

Passive surveillance for the early detection of notifiable avian influenza is based on clinical signs (*12*), an anamnesis of exponentially increasing death in the affected flock, or both. This surveillance is effective for acute infection causing severe disease (mainly HPAIV infection) but less so for LPAIV infection, which often causes mild or no disease. Samples (blood, tissue, and/or tracheal and cloacal swabs) of diseased/dead birds are tested by ELISA, PCR, and virus isolation.

Early warning includes signals such as aberrations in production parameters (decreased egg production, increased death rates, decreased feed and/or water intake). It excludes avian influenza as the cause of clinical problems in poultry flocks in situations in which birds show clinical signs that can be caused by other avian pathogens. Tracheal and cloacal swabs are tested for avian influenza by PCR (exclusion diagnostics).

The serologic monitoring program is active surveillance to detect all avian influenza virus incursions, even those that remain subclinical. This program is much more intense than required by the European Union: all poultry farms, except outdoor-layer farms and turkey farms, are tested at least once a year. Thirty samples per farm are screened by ELISA, and positive samples are confirmed by hemagglutination-inhibition test. Outdoor-layer farms are tested 4 times per year, and turkey farms are tested each production cycle. Meat-turkey farms have an average production cycle of ≈4 months; for broilers and meat ducks, this cycle is 5–6 weeks. All sampling is done just before slaughter, except the 3 extra samplings in outdoor-layer farms.

Farms were identified by their unique farm number and categorized on the basis of poultry production type (PT): duck breeders, meat ducks (meat production), turkey breeders, meat turkey, broilers, broiler breeders, indoor-layers, outdoor-layers, and layer breeders.

We selected putative spatial-environmental risk factors for LPAIV introduction related to farm location for incorporation in the risk model. These risk factors were distance to clay soil, distance to waterways, and distance to defined wild waterfowl areas. 

We analyzed the farms’ distance to clay soil (Geodesk database [GDB3]; Wageningen University, Wageningen, the Netherlands). Clay soil is a sediment of large rivers and is, in epidemiologic terms, a proxy for the presence of large water quantities, which is a proxy for an attractive environment for wild waterfowl. Wild waterfowl is presumed to be the most important reservoir for LPAIV. Presence of clay soil close to poultry farms was a risk factor for LPAIV introduction on outdoor-layer farms (*13*).

We also assessed distance from farms to waterways. Three sizes of waterways (width in meters) were included in the model: small (0.5–3 m wide), medium (3–6 m wide), and large (>6 m wide). Presence of waterways is a proxy for an attractive environment for wild waterfowl; spatial data of waterways was available from the Dutch Land Registry (http://www.kadaster.nl/web/artikel/producten/TOP10NL.htm).

Distance to defined wild waterfowl areas is a direct proxy for a possible avian influenza virus reservoir. Wild waterfowl areas were defined as follows: areas with on average >5 wild water birds counted per hectare (based on systematic regular bird census schemes by Sovon [Nijmegen, the Netherlands], which coordinates the monitoring of wild bird populations in the Netherlands). Birds of the families *Anatidae*, *Laridae*, and *Rallidae* were included; these birds are known avian influenza virus carriers (*14*,*15*) (Technical Appendix).

### Positive Farms

Positive farms were defined as follows: farms with >1 seropositive animal to any avian influenza strain in both the screening ELISA (IDEXX FLockCheck AI MultiS-Screen, IDEXX Europe B.V., Hoofddorp, the Netherlands) and the confirmatory hemagglutination-inhibition test; or farms with >3 positive results (of 30 serum samples) in the screening ELISA. Furthermore, we included in the analysis only primary cases (excluding secondary spread detected by epidemiologic tracing).

### Period at Risk

#### Positive Farms

For every year, we estimated the period at risk (in months) as the sum of the period from January 1 and the last negative sampling plus half of the period between the last negative sampling and the positive sampling. In case of no negative sampling in the year the farm became positive, the last negative sampling of the year before was included. In that instance, the time at risk was estimated as half of the period from the last negative sampling to the first positive sampling. Broilers, meat turkeys, and meat ducks were sampled 1 week before the end of their production. Therefore, the period at risk for these PTs was set at a fixed period.

#### Negative Farms

For every year, we estimated the period at risk (in months) as the period from January 1 through last negative sampling. This sampling was done for all PTs except broiler, meat-turkey, and meat-duck farms. For the latter, the period at risk was the same as for the corresponding positive farms.

### Statistical Analysis

We analyzed data using the statistical software R version 3.1.3 (https://www.r-project.org/). The relative risk (RR) of introduction of LPAIV per type of poultry farm (PT), during the study period (2007–2013) was quantified using multivariate statistical models (known as generalized linear models or generalized linear mixed models [GLMMs]) (Technical Appendix). We used indoor-layer chicken farms as the reference category. In terms of disease causation, if the RR is <1, the factor is considered a sparing factor, whereas if the RR is >1, the factor is considered a putative causal factor (*16*). In addition, we studied the effect of the spatial-environmental variables (distance to clay soil, waterways, and wild waterfowl areas) on the risk for LPAIV introduction. Statistical investigation started with a univariate analysis; distance of clay soil to the location of poultry farms was significantly associated with risk for LPAIV introduction only for layer (indoor and outdoor) farms. The different categories of waterways were significantly associated with risk for LPAIV introduction, but medium-sized waterways showed by far the strongest association. Thus, in the multivariate analysis, distance to clay soil and small- and large-sized waterways fell out of the model in the selection process; distance to medium-sized waterways and distance to wild waterfowl areas were strongly associated with risk for LPAIV introduction and stayed in the model when tested together in the multivariate analysis.

## Results

**Figure 1:**
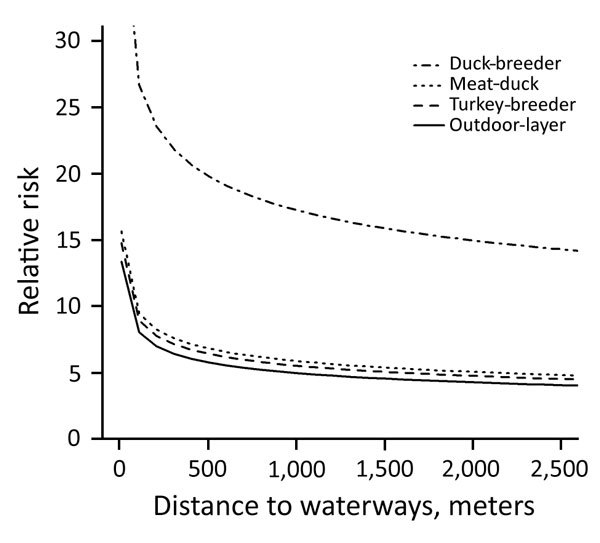
Risk for introduction of low pathogenicity avian influenza virus into duck-breeder, meat-duck, meat-turkey, and outdoor-layer farms, the Netherlands, 2007–2013. For the estimation of the relative risk as a function of distance to medium-sized waterways (3–6 m wide), distance to wild waterfowl areas was kept constant.

**Figure 2:**
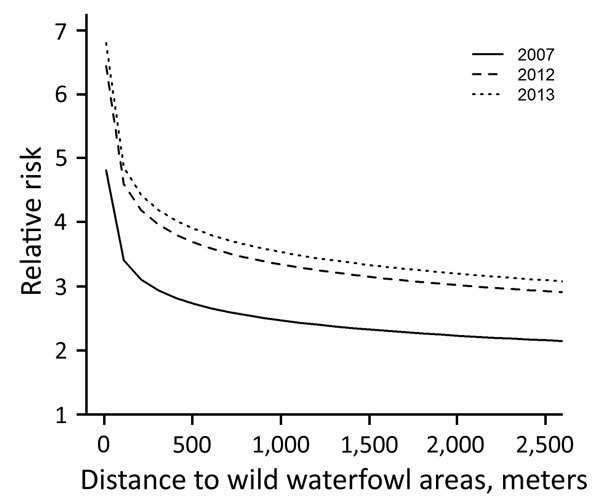
Risk for introduction of low pathogenicity avian influenza virus into outdoor-layer farms, the Netherlands, 2007–2013. Relative risk is shown for 2007 (reference for between-year comparison), 2012 (p = 0.08), and 2013 (p = 0.005). For the estimation of the relative risk as a function of distance to wild waterfowl areas, distance to medium-sized waterways (3–6 m wide) was kept constant.

**Figure 3:**
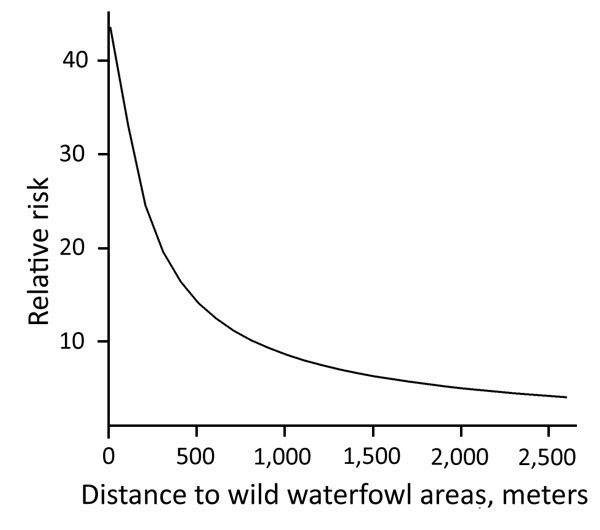
Relative risk for introduction of low pathogenicity avian influenza virus into meat-turkey farms, the Netherlands, 2007–2013. No difference in risk was observed between surveillance years. For the estimation of the relative risk as a function of distance to wild waterfowl areas, distance to medium-sized waterways (3–6 m wide) was kept constant.

During 2007–2013, we surveyed 19,274 farms and detected 295 LPAIV introductions (Table 1). The Netherlands has a small population of turkey and duck breeder farms, and these small populations, in particular turkey breeders (only 1 farm in 2013 and a maximum of 5 in 2007), made it difficult to evaluate potential interactions (e.g., between PT and distance variables) when modeling the risk for introduction. Therefore, we first made an overall quantification of the RR for each PT and included the year of surveillance as a random effect in a GLMM. Broiler, broiler-breeder, and layer-breeder farms were at significantly lower risk for LPAIV introduction (p<0.05) than were indoor-layer farms (e.g., broiler farms had on average a 5 times [1/0.2] lower risk for LPAIV introduction than did indoor-layer farms) (Table 2). By contrast, the risk was significantly higher for outdoor-layer, duck, duck-breeder, meat-turkey, and turkey-breeder farms (p<0.05) (e.g., outdoor-layer farms had on average a 6.3 times higher risk for LPAIV introductions than indoor-layer farms). The effect of distance from medium-sized waterways to farm location was comparable for the different PTs, and we included this variable in the GLMM (Table 2). The risk for LPAIV introduction decreased with increasing distance from poultry farms to medium-sized waterways; RR was highest within the closest 500 m (Figure 1). To evaluate potential statistical interactions, we combined meat-turkey and turkey-breeder farms (which had similar RR estimates in our first analysis [Table 2]), and we evaluated the effect of the location variables and potential interactions. A generalized linear model fit better than a GLMM. We identified significant interactions between 1) year of surveillance and indoor- and outdoor-layer farms and 2) distance to wild waterfowl areas and outdoor-layer farms or meat turkey farms. The analysis showed a yearly decrease in the RR for indoor-layer farms (Table 3), in contrast to an increased risk for outdoor-layer farms for 2012 and 2013 (Figure 2). The risk for LPAIV introduction in outdoor-layer and meat turkey farms decreased with increasing distance to areas with wild waterfowl (Figures 2, 3). No significant risk was found for distance to clay soil.

**Table 1:** LPAIV surveillance data collated from poultry farms, the Netherlands, 2007–2013*

Type of farm	No. farms positive	Total no, farms	Median time at risk, mo	Median distance to wild water bird areas, m	Median distance to medium- sized waterway, m†	Probability of introduction‡	RR§
Indoor-layer	60	5,600	7.3	4,227	769	0.001	1
Outdoor-layer	143	2,549	6.3	3,996	670	0.009	6.0
Layer-breeder	14	2,174	9.5	4,157	738	0.001	0.5
Broiler	2	5,409	1.2	3,292	576	0.000	0.2
Broiler-breeder	14	2,718	8.5	4,002	824	0.001	0.4
Meat-turkey	30	469	3.7	3,208	1,042	0.017	11.7
Turkey-breeder	2	18	5.7	2,035	659	0.019	13.1
Meat-duck	16	267	1.2	3,477	1,180	0.050	33.9
Duck-breeder	14	70	5.8	4,107	767	0.034	23.4

*LPAIV, low pathogenicity avian influenza virus; RR, relative risk. †Distance to clay soil and distance to small- and large-sized waterways also included in the multivariate analysis (data not shown). They did not have a significant effect on the risk for LPAIV introduction. Waterway sizes were defined as follows: small, 0.5–3 m wide; medium, 3–6 m wide; large, >6 m wide. ‡Unadjusted probabilities of LPAIV introduction per farm months at risk. §These are the unadjusted RR estimates obtained by dividing the unadjusted probabilities of LPAIV introduction of each type of poultry farm by that of indoor-layer farms.

**Table 2:** Relative risks for introduction of low pathogenicity avian influenza virus infection in different types of poultry farms, the Netherlands, 2007–2013

Type of poultry farm	Relative risk (95% CI)	p value
Indoor-layer	1.0 (reference)	
Outdoor-layer	6.3 (4.7–8.6)	<0.00001
Layer-breeder	0.5 (0.3–0.8)	0.008
Broiler	0.2 (0.1–0.8)	0.02
Broiler-breeder	0.4 (0.2–0.8)	0.004
Meat-turkey	12.0 (7.8–18.8)	<0.00001
Turkey-breeder	11.3 (2.8–46.2)	0.0008
Meat-duck	39.5 (22.6–69.1)	<0.00001
Duck-breeder	25.5 (14.2–45.9)	<0.00001
Natural logarithm*	0.8 (0.7–0.9)	0.00005

*Of distance to medium-sized waterways in meters, i.e., 3–6 m wide.

**Table 3:** Yearly relative risk for introduction of low pathogenicity avian influenza virus in indoor-layer farms, the Netherlands

Year	Relative risk (95% CI)
2007	1 (reference)
2008	0.65 (0.48–1.04)
2009	0.63 (0.28–0.84)
2010	0.41 (0.28–0.68)
2011	0.56 (0.44–0.70)
2012	0.5 (0.30–0.83)
2013	0.15 (0.04–0.27)

## Discussion

Our study shows that outdoor-layer, duck (breeder and meat), and turkey (breeder and meat) farms have a significantly higher RR for LPAIV introduction than do indoor-layer farms. The higher risk in outdoor-layer farms probably reflects their higher exposure to LPAIV from a contaminated environment. The presence of avian influenza in wild water birds and the frequency of direct or indirect contact between reservoir birds and poultry are risk components that enable transmission from wild birds to poultry. However, in addition to the higher introduction rate on outdoor-layer farms (this study) and the genetic relationship of wild bird strains and avian influenza outbreak viruses (*17*), no scientific data have been available that could support this assumption, although physical environmental factors, such as surface water availability and proximity to lakes and wetlands, have been suggested as drivers of HPAIV H5N1 outbreaks in poultry and wild birds (*18*,*19*).

We described a significant spatial-environmental relationship: the closer to waterways—a proxy for an attractive environment for wild waterfowl—and wild waterfowl areas a farm is located, in particular outdoor-layer farms, the higher the risk for LPAIV introduction. Although waterfowl and shorebirds are known to form the major natural reservoir and source of all known influenza A viruses (*14**,**20*,*21*), there is little direct evidence for transmission of avian influenza virus from (wild) birds to poultry. Two lines of evidence suggest that wild birds can be the source of avian influenza infection in poultry: 1) temporal associations between avian influenza virus isolated from wild birds and from outbreaks in poultry flocks and 2) genetic similarity between avian influenza virus strains isolated from wild birds and from poultry. Phylogenetic studies support the presumed transmission route from wild birds to poultry. For example, a LPAIV H7N7 caused the HPAI H7N7 epidemic in the Netherlands that started at a free-range farm (*22*). This virus is believed to be a reassortant of an H7N3 virus and an H10N7 virus isolated from mallards in 2000 during survey studies of migratory wild birds in the Netherlands (*23*). Furthermore, recent genetic analyses of HPAIV H5N8 strains from the Netherlands, and of other strains from countries in Europe, South Korea, and Japan, suggested that the strains from Europe probably arrived through migratory wild birds from Asia, most likely through overlapping flyways and common breeding sites in Siberia (*24*,*25*).

In the Netherlands, turkeys are raised indoors, and despite the small number of turkey farms, we observed a higher RR for introduction of LPAIV infection to breeder and meat-turkey farms. This higher risk might be associated partly with the apparent higher susceptibility of turkeys than chickens to LPAIV infection (*26*).

As reported by Gonzales et al. (*10*), we found that duck-breeder farms have the highest RR for LPAIV introduction. This risk could be related to their higher susceptibility to infection with LPAIV of wild water bird origin (ducks, geese, and swans) than chickens (*27*) and their long production cycle (time of exposure). We also observed a significantly higher risk for LPAIV introduction into meat-duck farms than into indoor-layer farms. This finding is somewhat surprising because meat ducks are kept indoors and have a short production cycle (6.5 weeks), in contrast with broilers, which also are kept indoors, have a short production cycle (6 weeks), and had a very low risk for LPAIV introduction. The higher susceptibility of ducks than chickens to LPAIV (*27*) could be a reason to explain this contrast. In addition, poor biosecurity compliance might play a role. For instance, floor bedding for ducks is stored outside (often not protected by a cover) and transported inside the duck house several times during the growing period. Bedding material for broilers is mostly stored inside the poultry house and is placed only once during the production cycle or not replaced. Poor biosecurity compliance has been reported repeatedly in poultry production (*28**–**30*). Meat ducks and broilers are tested before slaughter, and considering that the time to build up a serologic prevalence after an LPAIV infection that can be detected by random sampling could take ≈2–3 weeks (*31*), LPAIV introductions that occur shortly before slaughter could be missed. Therefore, the RRs could be underestimated for both meat ducks and broilers. Nevertheless, by looking at the large number of broiler flocks tested along these years, the fact that only 2 LPAIV introductions were detected, and the fact that surveillance was able to detect a relatively high number of LPAIV introductions in meat ducks (also short production cycle), we conclude that the risk for LPAIV introduction in broilers is low under housing conditions in the Netherlands.

In addition, the RR for layer-breeder farms was 5 times lower for LPAIV introduction than it was for indoor-layer farms (2011–2013). These findings might be related to the high biosecurity levels on these PTs.

Our finding that the RR for LPAIV introduction on outdoor-layer farms increased over time (a significantly higher RR in 2013 than in 2007, 2008, 2009, and 2011) can be explained by an increase of the number of introductions on outdoor-layer farms, especially in 2012 and 2013. An increase in the number of outdoor-layer farms and a decrease in the number of indoor-layer farms (for which RR decreased over time), particularly in 2012 and 2013, might partly explain these changes in risk. Further research is needed to gain insight into the factors that might affect introduction rates and differences over time. A plausible explanation might be increased direct or indirect contact between outdoor ranging poultry and infectious wild bird populations, but this explanation remains speculative because field data on the type and frequency of contact between wild birds and poultry in outdoor-layer farms is still missing. Climate and land use changes during the past decades have affected winter and breeding bird community composition (*32*); effects on herbivorous birds (such as many waterfowl species) through phenology-induced changes of plant forage quality and availability are most pronounced (*33*,*34*).

As recent experience shows, wild birds can introduce HPAIV directly into poultry (*24*,*25*), and HPAIV can emerge after an LPAIV H5/H7 introduction in poultry after varying lengths of time (*8*). If a notifiable LPAIV subtype infects a farm and later spreads to other farms before detection, the risk increases for mutation to HPAIV (*35*). Therefore, the sooner an introduction is detected, the sooner restrictive measures can be applied to contain the infection, ideally even to the index farm. Early detection and removal of infected poultry will help lower viral replication rounds.

Surveillance programs are important tools to prevent new HPAIV outbreaks. In the Netherlands the avian influenza surveillance program is much more intense than required by the European Union (*9*). Frequent sampling of high-risk poultry farms may help reduce the risk for transmission between farms (*31**,**36*). Based on expected risk factors for introduction, outdoor-layer farms (more contact with wild birds) and meat-turkey farms (higher susceptibility) are tested more frequently than other poultry farms. The results of our study indicate that duck farms also should be tested more frequently; passive surveillance will not easily detect LPAIV introductions in ducks because LPAIV will not cause observable clinical signs in them. Furthermore, it is clear that we should target surveillance not only toward PT, but also on location (e.g., within 500 m of waterways, wild bird areas, or both). In addition, there could be a discouraging strategy for settlement of new poultry farms in high-risk areas.

Technical Appendix Wild birds and multivariable statistical models used in the analyses
