# Supplementary material for: Risk for Low Pathogenicity Avian Influenza Virus on Poultry Farms, the Netherlands, 2007–2013
Source: Emerg Infect Dis. 2017 Sep;23(9):1510–6. doi: 10.3201/eid2309.170276 (PMC5572893; doi:10.3201/eid2309.170276)
Supplement: Technical Appendix — Wild birds and multivariable statistical models used in the analyses [file 17-0276-Techapp-s1.pdf]

Article DOI: https://doi.org/10.3201/eid2309.170276

Risk for Low Pathogenicity Avian Influenza Virus on Poultry Farms, the Netherlands, 2007–2013

Technical Appendix

Wild Birds included in the Analysis

The following birds of the families *Anatidae*, *Laridae* and *Ralidae* were included:

Gadwall (*Anas strepera*), wigeon (*Anas penelope*), northern shoveler (*Anas clypeata*), mallard (*Anas platyrhynchos*), (northern) pintail (*Anas acuta*), teal (*Anas crecca*), red-crested pochard (*Netta rufina*), mute swan (*Cygnus olor*), black swan (*Cygnus atratus*), Bewick’s swan (*Cygnus bewickii*), whooper swan (*Cygnus cygnus*), bar-headed goose (*Anser indicus*), tundra bean goose (*Anser serrirostris*), pink-footed goose (*Anser brachyrhynchus*), greylag goose (*Anser anser*), greater white-fronted goose (*Anser albifrons*), cackling goose (*Branta hutchinsii*), greater Canada goose (*Branta canadensis*), barnacle goose (*Branta leucopsis*), dark-bellied brent goose (*Branta bernicla*), Egyptian goose (*Alopochen aegyptiacus*), shelduck (*Tadorna tadorna*), pochard (*Aythya ferina*), tufted duck (*Aythya fuligula*), scaup (*Aythya marila*), eider (*Somateria mollissima*), common scoter (*Melanitta nigra*), goldeneye (*Bucepala clangula*), coot (*Fulica atra*), black-headed gull (*Croicocepalus ridibundus*), common gull (*Larus canus*), lesser black-backed gull (*Larus graellsii*), herring gull (*Larus argentatus*), and great black-backed gull (*Larus marinus*).

Multivariate Statistical Models Used in the Analyses

Data were analyzed using both generalized linear models (GLM) and generalized linear mixed models (GLMM), modeling the rate of introduction per time at risk (number of introductions per flock per time at risk) (*1*); both types of models used a binomial error distribution with a *cloglog* link and using log (time at risk in months) as an offset. The model was derived as follows: assuming that β is the rate of introduction of infection into a farm, then

the probability of infection p in a given time interval t is $1 – \exp(– \beta t)$. Following this reasoning, we modeled p as $p_{ij} = 1 – \exp(– \beta_j t_{ij} y)$, which upon linearization gives $\log(-\log (1 – p_{ij})) = \log \beta_j + \log t_{ij} + \log y$. In this model, the status of farm i of poultry type j (p_{ij}) is the binary response variable, $\log \beta_j$ is the vector of regression coefficients of the explanatory variables production type and the spatial-environmental variables (distance to clay soil, waterways, and wild waterfowl) and possible interactions between these variables (production type [PT] × spatial-temporal) "time at risk" in months ($\log t_{ij}$) is the offset, and $\log y$ (year of surveillance) is the grouping variable (random effect) when fitting a GLMM, or an explanatory variable when fitting a GLM. Indoor-layer chickens were the reference category; therefore, the exponent of the model intercept $\log \beta_0$ represents the rate of introduction of low pathogenicity avian influenza virus into indoor-layer chicken farms per month at distance zero from the spatial-environmental variables. For a different PT, this rate is the exponent of the sum of $\log \beta_0$ and the corresponding regression coefficient $\log \beta_j$. The exponent of $\log \beta_j$ of each PT was interpreted as the relative risk of introduction of low pathogenicity avian influenza virus. The fit of the model was assessed by residual analysis. The GLMM were fitted using the library lme4. Continuous variables (distance to clay soil, waterways, and wild waterfowl areas) were logarithm transformed, to ensure their linear relationship with the risk for introduction. Linearity of variables used in the model was further tested by introducing spline terms in the statistical models.

Reference

1. Gonzales JL, Elbers ARW, Bouma A, Koch G, de Wit JJ, Stegeman JA. Low-pathogenic notifiable avian influenza serosurveillance and the risk of infection in poultry—a critical review of the European Union active surveillance programme (2005–2007). Influenza Other Respir Viruses. 2010;4:91–9. http://dx.doi.org/10.1111/j.1750-2659.2009.00126.x. **PMID: 20167049**
